# Central relay of bitter taste to the protocerebrum by peptidergic interneurons in the *Drosophila* brain

**DOI:** 10.1038/ncomms12796

**Published:** 2016-09-13

**Authors:** Sebastian Hückesfeld, Marc Peters, Michael J. Pankratz

**Affiliations:** 1 Department of Molecular Brain Physiology and Behavior, Life and Medical Sciences Institute (LIMES), University of Bonn, 53115 Bonn, Germany

## Abstract

Bitter is a taste modality associated with toxic substances evoking aversive behaviour in most animals, and the valence of different taste modalities is conserved between mammals and *Drosophila*. Despite knowledge gathered in the past on the peripheral perception of taste, little is known about the identity of taste interneurons in the brain. Here we show that hugin neuropeptide-containing neurons in the *Drosophila* larval brain are necessary for avoidance behaviour to caffeine, and when activated, result in cessation of feeding and mediates a bitter taste signal within the brain. Hugin neuropeptide-containing neurons project to the neurosecretory region of the protocerebrum and functional imaging demonstrates that these neurons are activated by bitter stimuli and by activation of bitter sensory receptor neurons. We propose that hugin neurons projecting to the protocerebrum act as gustatory interneurons relaying bitter taste information to higher brain centres in *Drosophila* larvae.

## Abstract (web summary)

Bitter taste evokes aversive behaviour in animals, but little is known about the central nervous system mechanisms that convey this taste modality. Hückesfeld *et al*. identify a set of second order neurons in *Drosophila* that contain hugin neuropeptide and are responsible for conveying bitter taste to the protocerebrum.

## Graphical abstract

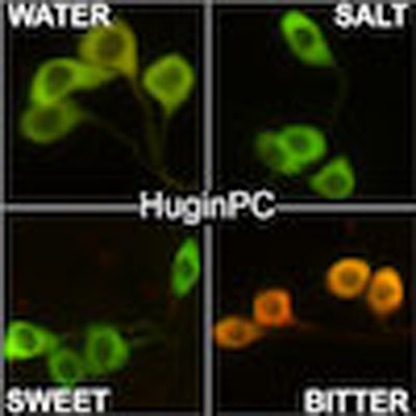

Detailed knowledge exists on the anatomical distribution and function of gustatory receptors in mammals and *Drosophila*^1^,^2^,^3^,^4^,^5^. In *Drosophila* 60 gustatory receptor genes encode 68 gustatory receptors^6^,^7^,^8^, with the majority of these receptors detecting bitter compounds^9^. Although gustatory receptors in *Drosophila* share no homology to mammalian taste receptors, the strategy used in both to detect a taste molecule, process its information and the valence of aversive bitter and appetitive sweet stimuli share similarities^4^. In contrast to the extensive knowledge on the peripheral coding of taste in flies and mammals, much less is known about the central pathways that relay and translate these signals into meaningful behaviour. Although broad regions in different parts of the brain have been shown to respond to various taste cues, there is little information on the molecular identity of specific neurons that convey different taste modalities to the higher brain centres^10^,^11^. Recently, secondary neurons that relay sweet taste from subesophageal zone (SEZ) to the antennal mechanosensory motor centre of adult *Drosophila* were characterized^12^. Analogous secondary neurons for other taste modalities have not yet been identified.

A candidate for conveying bitter taste from the SEZ to higher brain centres are neurons that express the hugin neuropeptide^13^,^14^ (referred to as hugin neurons), whose arborizations in *Drosophila* larvae overlap with that of bitter gustatory receptor neurons (GRNs) expressing the caffeine receptor GR66a^15^,^16^,^17^. In adult *Drosophila*, GR66a was shown to represent a bitter receptor for detection of caffeine^9^,^16^,^17^ and inactivation of GR66a positive neurons leads to impairment of caffeine aversion^17^. In *Drosophila* larvae, artificial activation of GR66a positive neurons leads to aversive behaviour^18^. Thus, hugin neurons were good candidates for acting as a central relay for bitter information from sensory neurons.

Using classical two-choice behavioural experiments, electrophysiological measurements as well as calcium imaging analysis, we now show that hugin neurons relay caffeine as well as other bitter taste signals from sensory neurons to the protocerebrum, acting as bitter taste interneurons in the *Drosophila* larval brain.

## Results

### Hugin neurons are required for caffeine avoidance response

**Figure 1:**
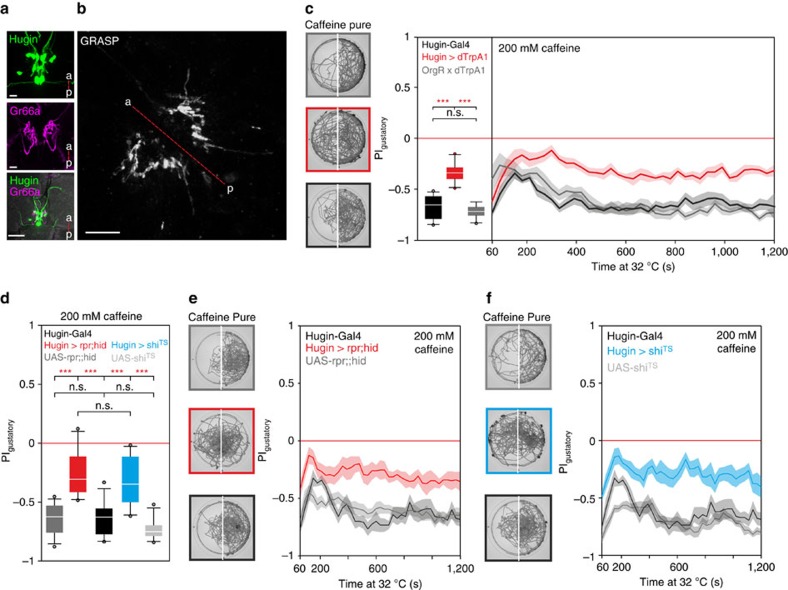
Hugin neurons are part of bitter gustatory pathway. (**a**) Expression analysis of hugin neurons and GR66a positive dendrites in the SEZ using Hug-YFP;UAS-mRFP line crossed to GR66a-Gal4. Scale bars, 10 μm for upper two panels and 50 μm for lowest panel. (**b**) Close proximity of GR66a positive dendrites and hugin positive dendrites located in the SEZ using GRASP (Hug1.2lexA attp40 driving lexAop-CD4::spGFP11 crossed to GR66a-Gal4 driving UAS-CD4::spGFP1-10). Scale bar, 10 μm. a—P=anterior—posterior. (**c**) Two-choice assay with 200 mM caffeine. Left representative plates are time projections of the last 5 min of the 20 min experiment. Activating hugin neurons with UAS-dTrpA1 (*n*=10) leads to impairment of choice behaviour (*P*<0.001, compared with the controls OrgR × dTrpA1 (*n*=10) and Hugin-Gal4 (*n*=11), Mann-Whitney-U-Rank-Sum-Test (MWU-TEST)). Controls did not significantly differ from each other (*P*=0.275, MWU-TEST). (**d**–**f**) Two-choice assay with 200 mM caffeine. Ablation of hugin neurons by expression of UAS-rpr;;hid (*n*=10) leads to impairment of bitter substrate avoidance compared with HugS3-Gal4 (*n*=10) and UAS-rpr;;hid (*n*=14) controls (*P*<0.001, MWU-Test). Controls did not differ from each other (*P*=0.728, MWU-Test). Silencing hugin neurons by expression of UAS-shibire^TS^ (*n*=10) shows same impairment on gustatory bitter choice compared with HugS3-Gal4 (*n*=10) and UAS-shi^TS^ (*n*=10) controls (*P*<0.001, MWU-Test). Controls did not differ from each other (*P*=0.168, UAS controls *P*=0.082, MWU-Test). All two-choice experiments were performed at 32 °C. Boxplots were generated from *PI* values of the last 5 min of the 20 min experimental time. Significances are indicated as ***P<0.001, **P<0.01 and *P<0.05. Line plots showing the time course of the two choice experiments are displayed as mean (line)±s.e.m. (transparent areas). Details of descriptive statistics and statistics against chance levels for experimental lines are shown in Supplementary Table 1.

We first asked whether the hugin neurons make contacts with caffeine responsive GR66a neurons. Using the GFP reconstitution across synaptic partners (GRASP) approach^19^, we could indeed observe a GRASP signal in the SEZ, indicating that caffeine receptor neurons and hugin neurons are in close proximity to each other (Fig. 1a,b).

Activating the hugin neurons causes the larvae to stop feeding and move out of a strongly appetitive food source (yeast)^20^, which could be due to activation of an aversive bitter taste pathway. We therefore tested the behavioural response to caffeine in a two choice assay. When hugin neurons are activated with the temperature sensitive cation channel dTrpA1, the animals showed impaired aversion to caffeine stimuli (Fig. 1c). We next asked how the animals would behave if hugin neuronal activity was suppressed, by ablating the hugin neurons. These animals showed significantly less avoidance to caffeine (Fig. 1d,e). To exclude potential developmental effects, we also expressed the temperature sensitive mutant form of dynamin (*shibire*^TS^) in the hugin neurons, which leads to a block of synaptic release in a temperature dependent manner. The loss of proper bitter aversion still persisted (Fig. 1d,f).

**Figure 2:**
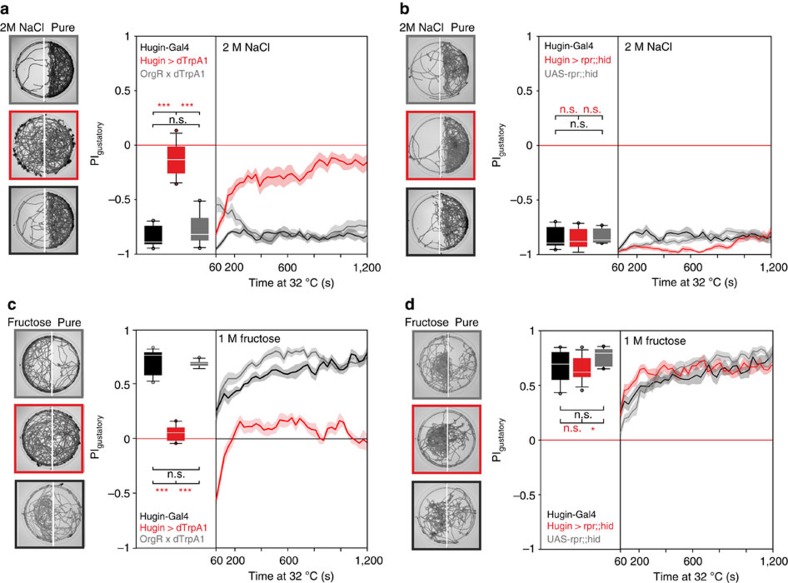
Hugin neurons are not required for high salt or sweet taste processing. (**a**) Two choice assay with 2 M NaCl. Activating hugin neurons with UAS-dTrpA1 (*n*=11) leads to impairment of choice behaviour (*P*<0.001 compared with OrgR × dTrpA1 (*n*=10) and Hugin-Gal4 (*n*=10) controls, Mann-Whitney-U-Rank-Sum-Test (MWU-Test)). Controls did not differ from each other (*P*=0.241, MWU-Test). (**b**) No significant difference in avoidance behaviour was observed on 2 M NaCl between Hugin>rpr;;hid (*n*=10) and UAS-rpr;;-hid control (*n*=10), (*P*=0.165, MWU-Test) or Hugin-Gal4 control (*n*=10), (*P*=0.838, MWU-TEST). Controls did not differ from each other (*P*=0.241, MWU-Test). (**c**) Two choice assay with 1 M fructose. Activating hugin neurons with UAS-dTrpA1 (*n*=11) leads to impairment of choice behaviour (*P*<0.001 compared with OrgR × dTrpA1 control (*n*=11) and Hugin-Gal4 control (*n*=13), MWU-Test). Controls did not differ from each other (*P*=0.384, MWU-Test). (**d**) There was no significant difference on 1 M fructose choice behaviour between Hugin>rpr;;hid (*n*=12) and Hugin-Gal4 control (*n*=10) (*P*=0.668, MWU-Test). UAS-rpr;;hid control larvae (*n*=10) showed significant difference to Hugin>rpr;;hid (*P*=0.013, MWU-Test). Controls did not differ from each other (*P*=0.104, MWU-Test). Sample two choice plates are shown on the left side of each experiment for each genotype for the last 5 min of the experiment. Boxplots were generated from *PI* values of the last 5 min of the 20 min experiment. Significances are indicated as ***P<0.001, **P<0.01 and *P<0.05. Line plots showing the time course of the two choice experiments are displayed as mean (line)±s.e.m. (transparent areas). Details of descriptive statistics and statistics against chance levels for experimental lines are shown in Supplementary Table 2.

**Figure 3:**
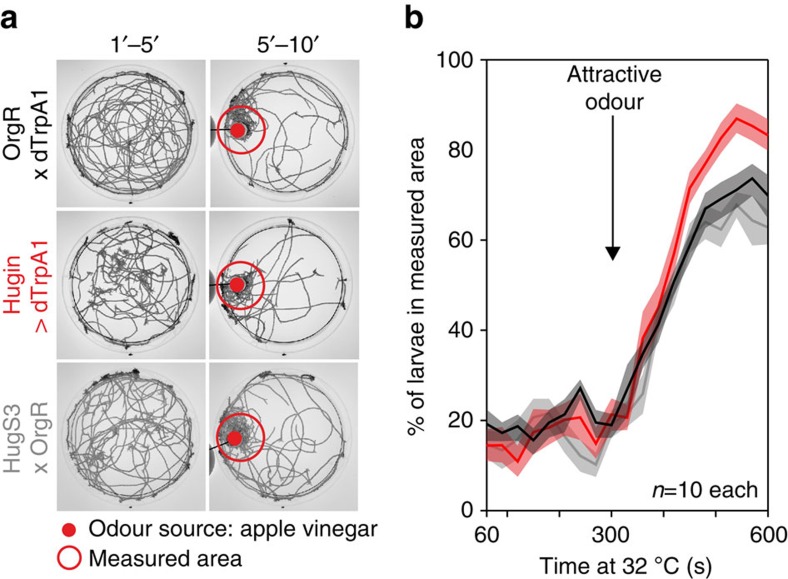
Activation of hugin neurons does not impair attractive olfactory guidance. (**a**) Olfactory assay for choice behaviour in response to an attractive odour (apple vinegar). Shown are time projections of the pure agar plates for the first 5 min of the experiment and the last 5 min of the experiment with the appetitive odour source apple vinegar. (**b**) Larvae of all three geneotypes (OrgR × dTrpA1, Hugin>dTrpA1 and HugS3 × OrgR) detected the appetitive odour at the same timepoint and were equally fast at reaching the nearby area beneath the apple vinegar spot above the plate.

We next asked if hugin neurons were involved in other taste modalities. We first tested high salt (2 M NaCl), which is very aversive for larvae^21^, in two-choice assays. As with caffeine, activation of the hugin neurons led to animals showing impaired aversion to high salt (Fig. 2a). However, no difference to control was observed when hugin neurons were ablated, indicating that hugin neurons are not required for the behavioural response to high salt (Fig. 2b). High caffeine and salt levels are both aversive gustatory stimuli. To determine how manipulation of hugin neuronal activity affected the response to an appetitive cue, we performed two-choice assays with 1 M fructose. In this case, activation led to impaired attraction to fructose, whereas ablation had no effect relative to control (Fig. 2c,d; Supplementary Fig. 1). Thus, when hugin neurons are activated, the larvae are no longer able to respond properly to different taste modalities, such as yeast protein^20^, fructose, high salt and caffeine. This chemosensory response was specific for taste, as olfactory behaviour was not affected (Fig. 3), showing that activation of hugin neurons selectively disrupts gustatory chemosensory processing. However, inhibiting the hugin neurons results in the inability to respond appropriately to caffeine, indicating that hugin neurons process bitter taste information. In sum, when hugin neurons are activated, larvae perceive all tested substrates (water, caffeine, salt and fructose) as bitter tasting. In contrast, when hugin neurons are inactivated, only the bitter substrate choice is affected since proper detection of bitter compounds no longer occurs.

### Distinct hugin neurons modulate taste and feeding behaviour

**Figure 4:**
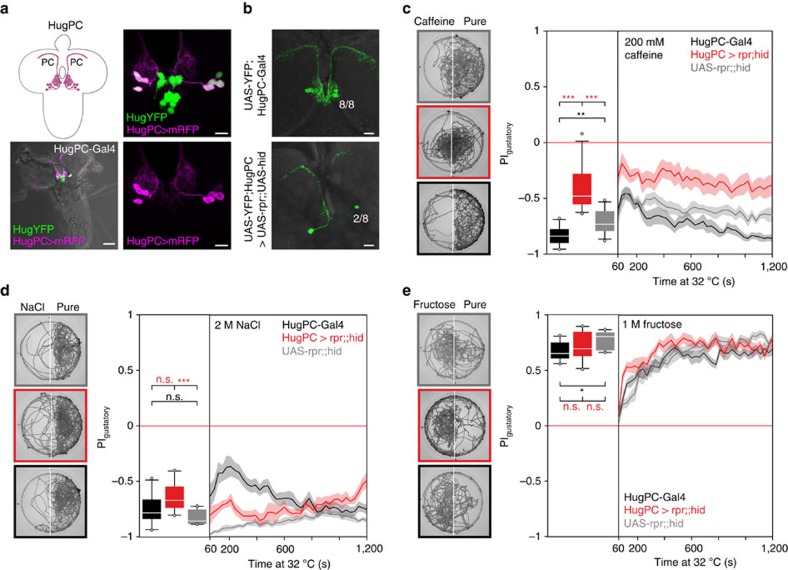
HuginPC neurons are necessary for bitter taste processing. (**a**) Expression of hugin neurons in HugPC-Gal4 line crossed to Hug-YFP;UAS-mRFP line. Eight hugin neurons that project to the protocerebrum are labelled by UAS-mRFP. Scale bars, 50 μm for left and 10 μm for right panels. (**b**) In HugPC>rpr;;hid larvae 6 of 8 huginPC neurons were ablated successfully (*n*=17); 2 (±0.3 SE). Scale bars: 20 μm. (**c**) Two choice assay with caffeine after ablation of huginPC neurons with UAS-rpr;;hid (*n*=13), (*P*<0.001 compared with HugPC-Gal4 control (*n*=10) and UAS-rpr;;hid control (*n*=14), Mann-Whitney-U-Rank-Sum-Test (MWU-Test)). Controls did differ from each other (*P*=0.006, MWU-Test). (**d**) Two-choice assay with 2 M NaCl. Ablating huginPC neurons with UAS-rpr;;hid (*n*=10) causes no impairment of high salt avoidance compared with HugPC-Gal4 control (*n*=10) (*P*=0.076, MWU-Test). A significant difference in salt avoidance occurred comparing HugPC>rpr;;hid with UAS-rpr;;hid larvae (*n*=10, *P*<0.001, MWU-Test). Controls did not differ from each other (*P*=0.167, MWU-Test). (**e**) Two-choice assay with 1 M fructose. Ablating huginPC neurons with UAS-rpr;;hid (*n*=10) causes no impairment in fructose attraction compared with HugPC-Gal4 control (*n*=10, *P*=0.241, MWU-Test) or UAS-rpr;;hid control (*n*=10, *P*=0.450, MWU-Test). Controls show significant difference to each other (*P*=0.013, MWU-Test). Boxplots were generated from PI values of the last 5 min of the 20 min experiment time in two-choice assays. Significances are indicated as ***P<0.001, **P<0.01 and *P<0.05. Line plot shows the time course of the two choice experiment displayed as mean (line)±s.e.m. (transparent areas). Details of descriptive statistics and statistics against chance levels for experimental lines are shown in Supplementary Table 3.

**Figure 5:**
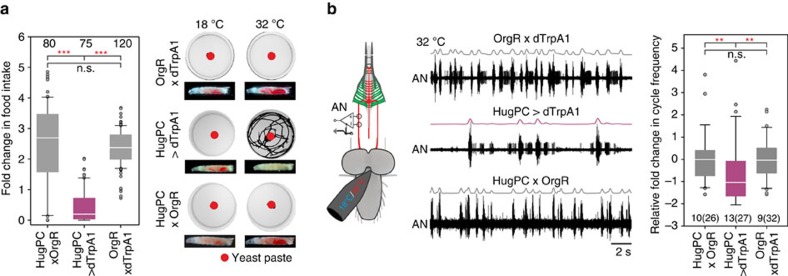
HuginPC neurons modulate feeding and wandering like behaviour. (**a**) HugPC-Gal4 line driving UAS-dTrpA1 (*n*=75 larvae). Larvae show reduction in food intake compared with OrgR × dTrpA1 (*n*=120 larvae) and HugPC × OrgR (*n*=80 larvae) (*P*<0.001, MWU-Test). Controls did not differ from each other (*P*=0.107, MWU-Test). Activation of huginPC neurons with dTrpA1 induces wandering like behaviour, where larvae leave the appetitive food source yeast. Shown are time projections over 20 min of plates with apple juice agar and a red spot of yeast in the middle. Decrease of food intake was measured as % of red stained gut content compared with the area of the whole larva. (**b**) Extracellular recordings of the antennal nerve (AN). Activation of huginPC neurons with UAS-dTrpA1 (*n*=13 larvae, 27 temperature steps) leads to significant decrease in cycle frequency of the AN motor pattern compared with OrgR × dTrpA1 control (*n*=9 larvae, 32 temperature steps) and HugPC × OrgR control (*n*=10 larvae, 26 temperature steps) (*P*=0.003, MWU-Test). Controls did not differ from each other. Significances are indicated as ***P<0.001, **P<0.01 and *P<0.05. Details of descriptive statistics are shown in Supplementary Table 4.

As the hugin neuronal cluster is composed of different classes with different projection targets^13^,^14^, we next wanted to determine which class was responsible for taste processing and feeding regulation. Through promoter deletion analysis, we generated a hugin promoter-Gal4 line which showed target gene expression exclusively in the eight hugin cells (four per hemisphere) that project to the protocerebrum (HugPC-Gal4, Fig. 4a). To see whether this subset of hugin neurons (huginPC neurons) is necessary for proper bitter taste processing, we ablated these neurons (Fig. 4b) and tested larvae in the caffeine two-choice experiment. Ablation of the huginPC neurons resulted in the inability to appropriately avoid caffeine as compared with control animals (Fig. 4c). Consistent with the ablation of all hugin neurons (Fig. 2), ablating just the huginPC neurons had no effect compared with controls on high salt or fructose (Fig. 4d,e, controls are re-plotted from Fig. 2b,d). We note that, in the case of 2 M NaCl, there was a significant difference to one of the controls (UAS-rpr;;hid control larvae). Therefore, we additionally tested the animals on a lower, but still aversive salt concentration (500 mM), which showed that huginPC neurons are not necessary for proper high salt aversion (Supplementary Fig. 2, for descriptive statistics see Supplementary Table 7). Activating just the huginPC neurons was sufficient to suppress food intake, induce wandering-like behaviour (Fig. 5a; Supplementary Fig. 3), as well as decrease the motor pattern of pharyngeal muscle contractions (Fig. 5b). These results indicated that the hugin neurons responsible for feeding and caffeine taste mediated behaviours are those that project to the protocerebrum.

### Activity of huginPC neurons upon bitter stimulation

**Figure 6:**
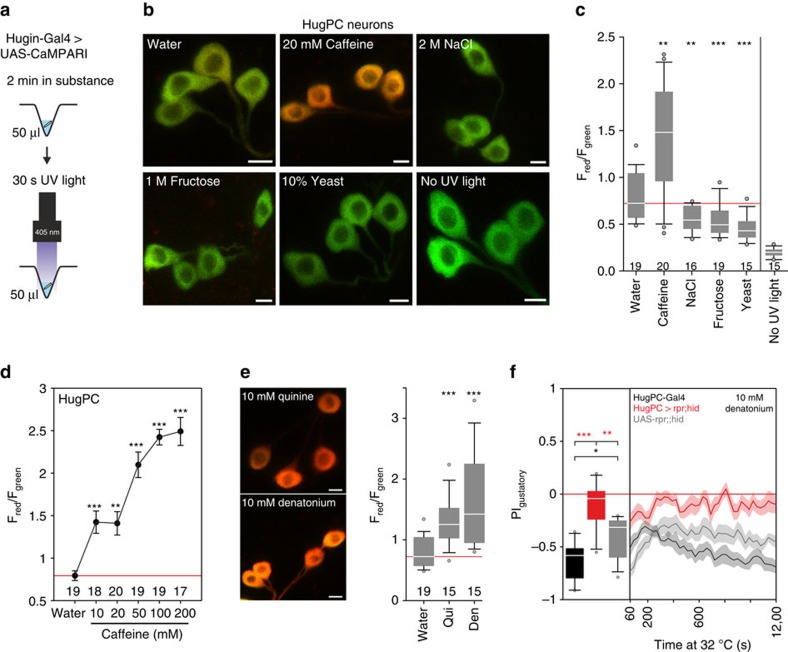
HuginPC neurons relay bitter taste information. (**a**) Experimental setup (left panel). (**b**) Larvae expressing UAS-CaMPARI in huginPC neurons showed green to red photoconversion only when larvae were placed in caffeine solution. Scale bars, 5 μm. (**c**) Quantification of F_red_ divided by F_green_ for *z*-projections of huginPC neurons (*P*=0.003 for caffeine, *P*=0.004 for NaCl, *P*<0.001 for fructose and *P*<0.001 for yeast mixed in water compared with pure water, Mann-Whitney-U-Rank-Sum-Test (MWU-Test), *n* of larvae displayed beneath boxplots). Right most boxplot shows fluorescence ratio of huginPC neurons without ultraviolet light exposure (*P*<0.001, MWU-Test). (**d**) Measurement of huginPC neurons expressing UAS-CaMPARI in larvae confronted with different concentrations of caffeine. HuginPC neurons display increasing red/green ratio with increasing caffeine concentration (10 mM: *P*<0.001, 20 mM: *P*=0.003, 50 mM: *P*<0.001, 100 mM: *P*<0.001, 200 mM: *P*<0.001 compared with water alone, MWU-Test). Dots represent mean, whiskers represent s.e.m. (**e**) HuginPC neurons expressing UAS-CaMPARI display high calcium activity in 10 mM quinine (*P*<0.001, MWU-Test) or 10 mM Denatonium (*P*<0.001, MWU-Test). numbers beneath boxplots indicate number of larvae used for each experiment. Scale bars: 5 μm. (**f**) Two-choice assay with 10 mM denatonium. Ablating huginPC neurons with UAS-rpr;;hid (*n*=10) leads to impairment of gustatory choice on denatonium compared with HugPC-Gal4 control (*n*=10, *P*<0.001, MWU-Test) or UAS-rpr;;hid control (*n*=12, *P*=0.002, MWU-Test). Controls show significant difference to each other (*P*=0.027, MWU-Test). Boxplots were generated from *PI* values of the last 5 min of the 20 min experiment time for two-choice experiment. Significances are indicated as ***P<0.001, **P<0.01 and *P<0.05. Line plots showing the time course of the two choice experiment and dot plots are displayed as mean (line)±s.e.m. (transparent areas). Details of descriptive statistics and statistics against chance levels for experimental lines are shown in Supplementary Table 5.

To further investigate the connection between bitter taste and hugin neurons, we asked if the huginPC neurons could be activated by caffeine using CaMPARI (Calcium Modulated Photoactivatable Ratiometric Integrator), that allows monitoring of calcium activity in intact animals. Calcium activity and simultaneous presence of ultraviolet-light (405 nm) lead to an irreversible conversion from green to red fluorescence of the neurons of interest^22^. When we placed intact larvae in solutions containing water and water mixed with caffeine, fructose, high NaCl or yeast (Fig. 6a), huginPC neurons were strongly activated by caffeine (Fig. 6b,c). They also showed a concentration dependent increase in calcium activity with increasing caffeine concentrations (Fig. 6d). Other bitter tastants, such as quinine and denatonium, also activated huginPC neurons (Fig. 6e). We tested the behavioural relevance for this rise in calcium activity for denatonium in two-choice experiments, since denatonium led to robust avoidance behaviour in control larvae at 32 °C (experimental temperature). The calcium activity in huginPC neurons was behaviourally relevant since ablation of these neurons resulted in larvae with impaired aversion to the taste of denatonium (Fig. 6f) (no difference when tested against chance levels, see Supplementary Table 5). Unexpectedly, we observed decrease in huginPC calcium activity in larvae placed in high salt, fructose and yeast relative to water alone (Fig. 6c). Although the functional significance of this repression is not clear, it has been shown that bitter taste pathways can inhibit sweet pathways in adult^23^ and larval^24^ *Drosophila*, reflecting an interaction between pathways involving different taste modalities. This cross-regulation might be a general strategy to efficiently detect distinct taste modalities, since in case of the huginPC neurons, a sweet taste stimulus would inhibit the activity of bitter interneurons.

**Figure 7:**
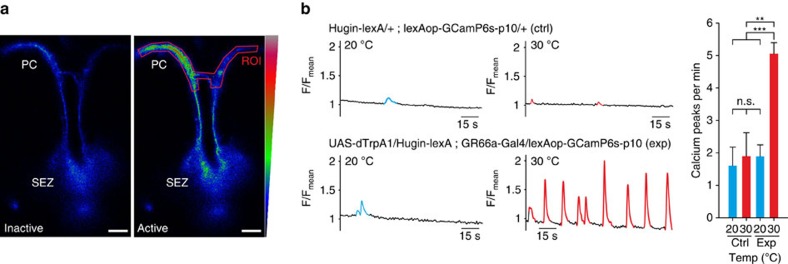
HuginPC neurons are functionally connected to GR66a neurons. (**a**) Example of huginPC neuronal arborizations in the protocerebrum expressing GCamP6s in inactive and active state. Scale bars, 20 μm. (**b**) Activation of GR66a neurons by dTrpA1 in larvae expressing Hugin-lexA-lexAop-GCamP6s. Sample traces of calcium currents show rhythmic activity of huginPC projections when GR66a neurons are activated at 30 °C. Quantification of calcium spikes per min showed no significant difference between control (ctrl, *n*=13) and experimental genotypes (exp, *n*=14) at 20 °C (*P*=0.241, Mann-Whitney-U-Rank-Sum-Test (MWU-Test); control at 20 and 30 °C (*n*=13), (*P*=0.790, MWU-Test); and control at 30 °C and experimental genotype at 20 °C (*P*=0.197, MWU-Test). Significant difference could be shown for the experiment at 20 and 30 °C (*n*=14) (*P*=0.004, MWU-Test), as well as control at 20 °C compared with experiment at 30 °C (*P*<0.001, MWU-Test). Scale bars, 20 μm. Significances are indicated as ***P<0.001, **P<0.01 and *P<0.05. Details of descriptive statistics are shown in Supplementary Table 6.

Finally we asked if there is a functional connection, in addition to the anatomical connection (Fig. 1a,b), between the caffeine sensing GR66a neurons and hugin neurons. To this end, we activated the GR66a neurons and then monitored the activity of hugin neurons using the calcium indicator GCaMP6s (Fig. 7a,b). Activation of GR66a by dTrpA1 and simultaneous calcium imaging of huginPC projections resulted in the induction of rhythmically occurring calcium peaks, demonstrating a functional connection between GR66a and huginPC neurons (Fig. 7b).

## Discussion

**Figure 8:**
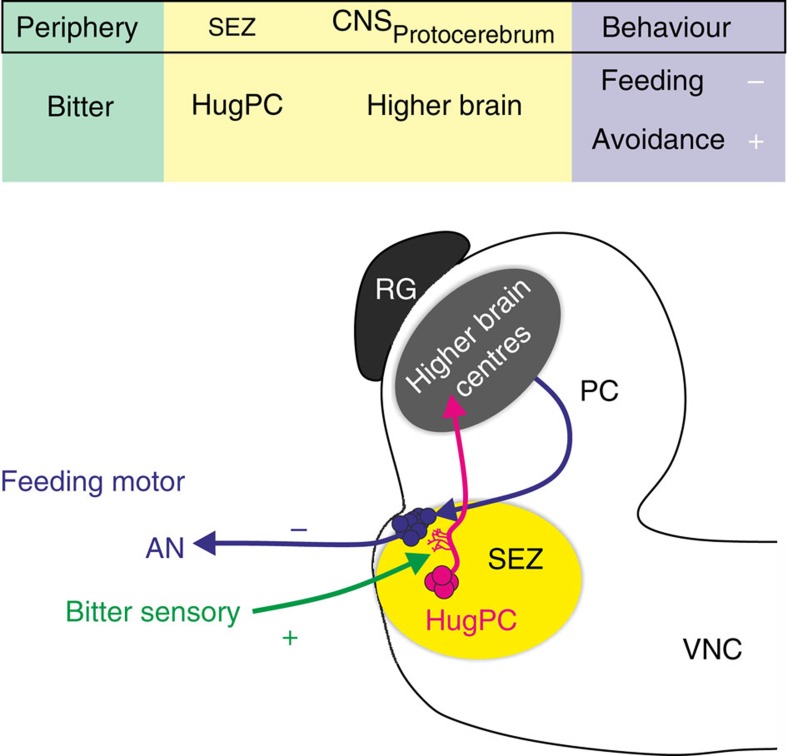
HuginPC neurons act as gustatory interneurons for bitter taste. Bitter taste, detected in peripheral taste organs, activates huginPC neurons. Activity of huginPC neurons projecting to higher brain centres leads to a decrease in food intake and in the cycle frequency of feeding related motor patterns in the antennal nerve (AN). Activation of huginPC neurons also leads to aversion to different gustatory substrates, including yeast, thereby triggering aversive behaviour. Thus, huginPC neurons act as relay of bitter taste to the protocerebrum in the *Drosophila* larval brain.

The bitter taste rejection response is important for all animals that encounter toxic or harmful food in their environment. Here we showed that the hugin neurons in the *Drosophila* larval brain function as a relay between bitter sensory neurons and higher brain centres (Fig. 8). Strikingly, activation of the hugin neurons made the animals significantly more insensitive to substrates with negative valence like bitter (caffeine) and salt (high NaCl), as well as positive valence like sweet (fructose). In other words, when the hugin neurons are active these animals ‘think' they are tasting bitter and therefore become insensitive to other gustatory cues. This is in line with observations made in mice, in which optogenetically activating bitter cortex neurons caused animals to avoid an empty chamber illuminated with blue light. In this situation, although mice do not actually taste something bitter, they avoid the empty chamber since the bitter perception has been optogenetically induced in the central nervous system (CNS) and the mice ‘think' they are tasting a bitter substance^25^.

In our previous work, activation of all hugin neurons led to behavioural and physiological phenotypes such as decreased feeding, decrease in neural activity of the antennal nerve (AN), and induction of a wandering-like behaviour^20^. We have now pinpointed the neurons responsible specifically to those that project to the protocerebrum. These neurons not only respond to bitter stimuli, but also show a concentration dependent increase in calcium activity in response to caffeine. Dose dependent coding of bitter taste stimuli was previously shown to occur in peripheral bitter sensory neurons, where bitter sensilla exhibit dose dependent responses to various bitter compounds^9^. Larvae in which the huginPC neurons have been ablated still showed some avoidance to caffeine. Whether this implies the existence of other interneurons being involved in caffeine taste processing remains to be determined. Interestingly, the huginPC neurons are inhibited when larvae taste other modalities like salt (NaCl), sugar (fructose) or protein (yeast). This may indicate that taste pathways in the brain are segregated, but influence each other, as previously suggested^10^.

Bitter compounds may be able to inhibit the sweet-sensing response to ensure that bitter taste cannot be masked by sweet tasting food. This provides an efficient strategy for the detection of potentially harmful or toxic substances in food^26^,^27^. For appetitive tastes like fructose and yeast, bitter interneurons neurons like the huginPC neurons in the CNS may become inhibited to ensure appropriate behaviour to pleasant food. Salt is a bivalent taste modality, that is, low doses of salt drive appetitive behaviour, whereas high doses of salt are aversive to larval^21^,^28^ and adult^29^,^30^ *Drosophila*. Inhibition of huginPC neurons when larvae are tasting salt might be due to a different processing circuit for different concentrations of salt and the decision to either take up low doses or reject high doses.

Taken together, we propose that hugin neuropeptide neurons projecting to the protocerebrum represent a hub between bitter gustatory receptor neurons and higher brain centres that integrate bitter sensory information in the brain, and through its activity, influences the decision of the animal to avoid a bitter food source. The identification of second order gustatory neurons for bitter taste will not only provide valuable insights into bitter taste pathways in *Drosophila*, but may also help in assigning a potentially novel role of its mammalian homologue, Neuromedin U, in taste processing.

## Methods

### Fly lines

Wild type (OrgR) crossed to UAS-dTrpA1 (Bloomington #26263) served as control in Figs 1, 2, 3 and 5. Hugin-Gal4 (HugS3-Gal4 (ref. 13), Bloomington# 58769), GR66a-Gal4 (second Chr., gift from K.Scott^31^(formerly described as GR66C1)), GR66a-Gal4 (third Chr., Bloomington# 57670 used in Fig. 7b), Hugin-lexA (Hug1.2-lexA attp40 (ref. 32)), HugPC-Gal4 (see generation of this Gal4-line below), UAS-eNpHR-YFP (Bloomington# 41753, referred here as UAS-YFP in Supplementary Fig. 3, since this homozygous line together with HugPC-Gal4 was used as fluorescent marker only), UAS-CaMPARI (Bloomington #58761), UAS-rpr;;UAS-hid (UAS-rpr (Bloomington# 5823) crossed homozygous to UAS-hid^33^), UAS-shibire^TS^^34^, lexAop-CD4::spGFP11 and UAS-CD4::spGFP1-10 were gifts from K. Scott^19^, 13x LexAop2-IVS-GCamP6s-p10 (Bloomington #44274).

### Fly care

Adult flies and larvae were kept on 25 °C under 12 h light/dark conditions. For electrophysiological and food intake experiments 4 h egg collections were made on apple juice agar plates containing a spot of yeast–water paste. After 48 h, larvae were transferred into food vials containing lab standard fly food. For other experiments (two-choice, CaMPARI, GCamP) larvae were raised in vials containing standard fly food with a spot of yeast for 4 days. All larvae used for the experiments were 98±2 h old. Only feeding third instar larvae were used for the experiments.

### Generation of transgenic flies

For HugPC-Gal4 line, a 544 bp Hugin fragment 155 bp upstream of the ATG was amplified with primer1 (AAG GGT TTG GTT TAA TTT ATT TAT GTC ATA) and primer2 (GAG CCT GAT TAG GTC CCT GAT GTT TAA ACT T) and cloned into pCaSpeR-AUG-Gal4-X vector (Addgene plasmid 8,378. The construct was injected into w^[1118]^).

### Two-choice gustatory assay

To measure the preference index of larvae towards given appetitive or aversive substrates, 9 mm diameter petri dishes were filled with 20 ml warm water agar (2.125% Agar-Agar, Kobe). After 20 min of air drying half of the agar was cut away and discarded. Compounds (2 M NaCl, 1 M Fructose, 200 mM Caffeine, 10 mM Denatonium) were diluted in warm agar in the given concentration until the agar fluid was clear, and filled in the other half of the petri dish (10 ml). After air drying again for 20 min, petri dishes were prewarmed in the 32 °C incubator 1.5 h prior to the experiment. All two-choice experiments were performed at 32 °C for comparability between all genotypes. For each experiment 30 larvae were taken out of standard fly food and washed with tap water. Larvae were then placed on the water side of the two-choice dishes and videotaped for 20 min. Videos were processed with FIJI (ImageJ) and analysed using a custom written script for FIJI. Analysis of PI values started 60 s after the start of the experiment to ensure proper tracking of larvae due larval accumulation at the beginning by placing them on the pure agar side. PI_gustatory_ was calculated as (#larvae_substrate_−#larvae_water_)/#larvae_total_.

### One-choice olfactory assay

For testing response to an attractive odour (apple vinegar), a one-choice assay was performed. Agar plates were used (as described earlier for two-choice petri dishes) and placed into the incubator at 32 °C for 1.5 h. Larvae were videotaped at 32 °C for 5 min, and then an Eppendorf cup (1.5 ml) with filter paper soaked with apple vinegar was placed on one side of the water agar plate above the larvae such that they were not able to reach the odorant source. Movement of larvae was analysed using the custom made FIJI macro.

### Food intake assay

Apple juice agar plates were prepared with a spot of red yeast paste in the middle of the plate. Plates were then placed in incubators precooled to 18 °C or prewarmed to 32 °C for 2 h. After 30 min starvation, five larvae were transferred on top of the yeast paste of a plate and videotaped for 20 min. After 20 min of videotaping, larvae were transferred into a small cell strainer and washed with 60 °C hot water. Larvae were then transferred to glass slides for photo documentation and analysed with the open source software FIJI (ImageJ) and a custom written analysis macro, which calculated the percentage of the red stained surface of the body compared with the whole body of the larva. To calculate the fold change of food intake from 18 to 32 °C, the value of all 32 °C values was divided by the mean value of all 18 °C values.

### Electrophysiology

Third instar larvae were dissected in 35 mm petri dishes coated with 5 ml two-component silicone (Elastosil RT). Larvae were pinned down dorsal side up at the anterior and posterior end using 77 μm thick sharp etched tungsten needles. The larva was cut open longitudinally along the dorsal midline and the cuticle was pinned aside with 40 μm tungsten needles. Interior organs like fat body, intestine or salivary glands were removed except for the cephalopharyngeal skeleton and CNS with attached nerves of interest. Eye and leg imaginal discs were also removed. A transversal cut of the cuticle was performed beneath the CNS to reveal the AN. Nerves not needed for the respective recording were cut. A piece of thinned Parafilm was placed beneath the nerve of interest. This nerve was isolated from the surrounding solution with two adjacent jelly pools. Motor output of the AN was measured using custom made silver wire electrodes connected to a preamplifier (Model MA103, Ansgar Büschges group electronics lab). The preamplifier was connected to a four-channel amplifier/signal conditioner (Model MA 102, Ansgar Büschges group electronics lab). All recorded signals were amplified (amplification factor: 5,000) and filtered (bandpass: 0.1–3 kHz). Recordings were sampled at 20 kHz. Data were acquired with Micro3 1,401 A/D board (Cambridge Electronic Design) and Spike2 software (Cambridge Electronic Design).

### Calcium imaging with CaMPARI

High-power LED of 405 nm (Thorlabs, M405L2–UV (405 nm) Mounted LED, 1,000 mA, 410 mW) was driven with a LED controller (Thorlabs, LEDDB1 driven with 1000, mA) and positioned 18 cm above the solution with the larva. PCR plate of 96-well was filled with 50 μl containing the given taste stimuli in tap water. Larvae were placed into the solution for 2 min and then ultraviolet light was applied for 30 s.

Afterwards brains were dissected in PBS and mounted onto a Poly-L-lysine (Sigma, Lot # SLBG4596V) coated cover slide with a drop of PBS. All z-stacks of the HugPC neurons were aquired using a ZEISS LSM 780 Laser scanning microscope with LCI Plan-Neofluar 25 × /0.8 Imm Korr DIC M27. For quantification of green to red photoconversion maximum intensity projections of the acquired z-stacks were used and a portion of the cytoplasmatic region of each cell was analysed to obtain data for green and red fluorescence intensity. Red fluorescence intensity was divided by green fluorescence intensity to get F_red_/F_green_ ratio. A ‘no ultraviolet light' control was included to show that scanning of the CNS without being exposed to ultraviolet light does not convert green to red fluorescence.

### GRASP

Genotypes used for GFP reconstitution across synaptic partners (GRASP) method^19^ were: Hug1.2lexA;lexAop-CD4::spGFP11 and GR66a-Gal4;UAS-CD4::spGFP1-10. Larval CNS was dissected and stained with anti-mouse-GFP (Abcam, 1:500, secondary antibody was anti-mouse-Al488 (Invitrogen, 1:500)). Images were acquired using a ZEISS LSM 780 Laser scanning microscope with LCI Plan-Neofluar 25 × per 0.8 Imm Korr DIC M27.

### Calcium imaging with GCaMP

Freshly dissected CNS of feeding third instar larvae of the genotypes UAS-dTrpA1/Hug1.2lexA; GR66a-Gal4/lexAop-GCamP6s-p10 (experiment) and Hug1.2lexA/CyO; lexAop-GCamP6s/TM3, Sb (control) were placed with the SEZ region up on a Poly-L-lysine coated coverslide in a drop of saline. Coverslide was attached to a custom built heating device consisting of a 1.5 cm^2^ Peltier element for shifting the temperature from 20 to 30 °C by applying specific voltage values. Images were acquired with a Zeiss LSM 780 laser scanning microscope as time series with a speed of 781.96 ms (∼1.28 Hz) using a Zeiss LCI ‘Plan-Neofluar' 25 × per 0.8 Imm Korr DIC M27 objective dipped in the saline solution. Region of interest covered the complete HugPC neuronal ‘sprinkler-like' arborization pattern in the protocerebrum.

### Statistics

For comparison of two groups in the two choice assays Mann-Whitney-Rank-Sum-Test was used. Statistical data were acquired as cumulative *PI* values of the last 5 min of the 20 min experiment and displayed as boxplots. For food intake analysis 32 °C values (% of red yeast in gut relative to whole body) were divided by the mean of all 18 °C values to gather the fold change of food intake. Fold changes were then compared with the Mann-Whitney-Rank-sum-Test. For electrophysiological data cycle frequencies were analysed at 18 °C for 60 s and 32 °C for 60 s. The fold change was calculated between 18 and 32 °C. Per larva a maximum of four temperature steps could be applied to the CNS during recordings. All fold changes of one genotype were then compared with the Mann-Whitney-Rank-Sum-Test with the other genotypes. The relative fold change is displayed as subtraction of the mean of OrgR × dTrpA1 control values from the fold change values of all genotypes (OrgR × dTrpA1 set to 0). CaMPARI data were analysed by calculating the mean F_red_/F_green_ value of all eight HuginPC neurons of one larva to determine one mean F_red_/F_green_ value per larva. Values were then compared with the Mann-Whitney-Rank-Sum-Test to water control.

### Data availability

The authors declare that the data supporting the findings of this study are available within the article (and its Supplementary Information files), or available from the authors upon request.

## Additional information

**How to cite this article:** Hückesfeld, S. *et al*. Central relay of bitter taste to the protocerebrum by peptidergic interneurons in the *Drosophila* brain. *Nat. Commun.* 7:12796 doi: 10.1038/ncomms12796 (2016).

## Supplementary Material

Supplementary Information Supplementary Figures 1-3 and Supplementary Tables 1-7
