# Supplementary material for: Central relay of bitter taste to the protocerebrum by peptidergic interneurons in the Drosophila brain
Source: Nat Commun. 2016 Sep 13;7:12796. doi: 10.1038/ncomms12796 (PMC5027282; doi:10.1038/ncomms12796)
Supplement: Supplementary Information — Supplementary Figures 1-3 and Supplementary Tables 1-7 [file ncomms12796-s1.pdf]

Supplementary material: Hückesfeld et al.

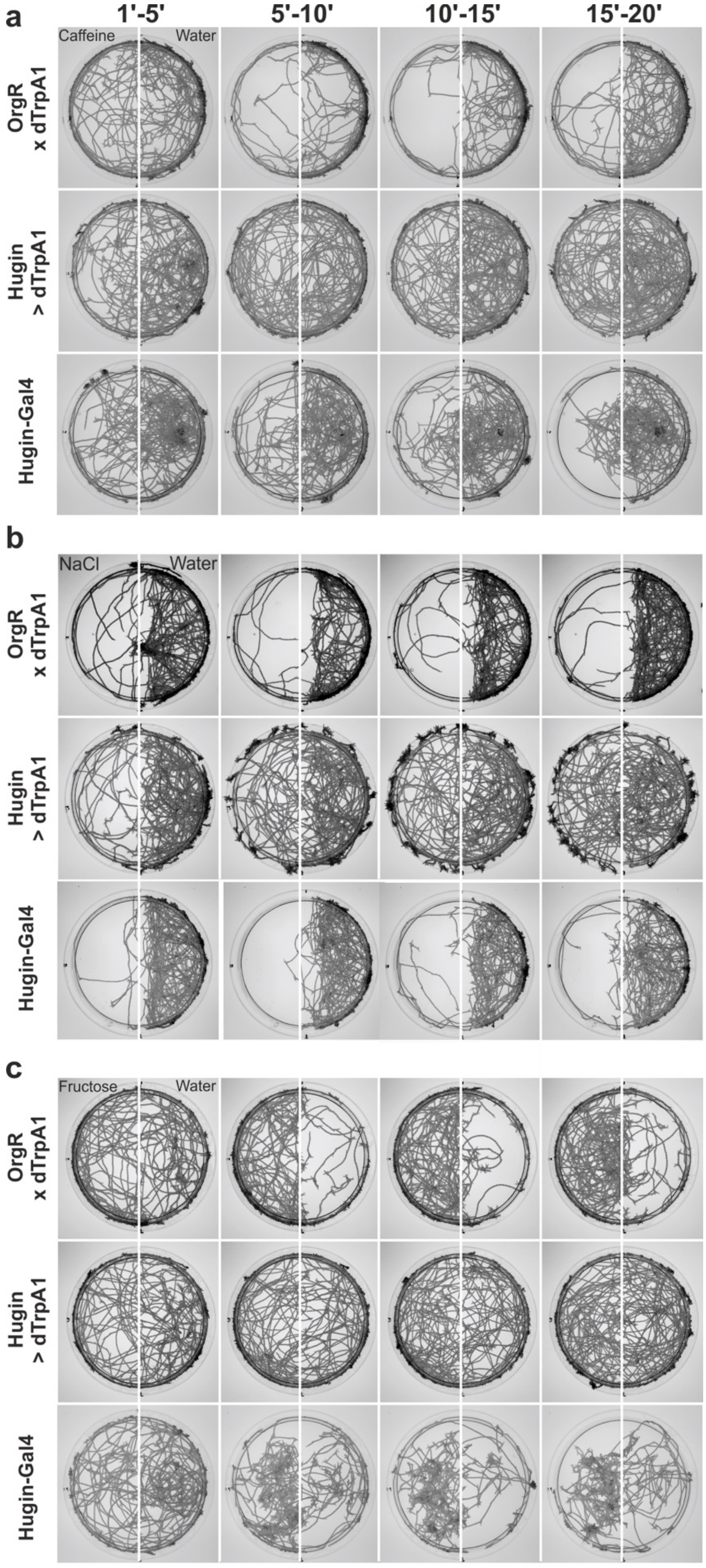

Supplementary Fig. 1: Attraction and aversion to taste substrates over time

Examples of 5 min time projections for each genotype in the 20 min two-choice experiments. **(a)** Two-choice plates with caffeine on the left side shown for Hugin-Gal4 line, Hugin > dTrpA1 and OrgR > dTrpA1. Hugin > dTrpA1 larvae show less aversion to the caffeine substrate than controls. **(b)** Hugin > dTrpA1 larvae show less aversion to the NaCl substrate than controls. **(c)** Hugin > dTrpA1 larvae show less attraction to the fructose substrate. Activation of Hugin neurons causes general loss of appropriate substrate choice for aversive and attractive substrates.

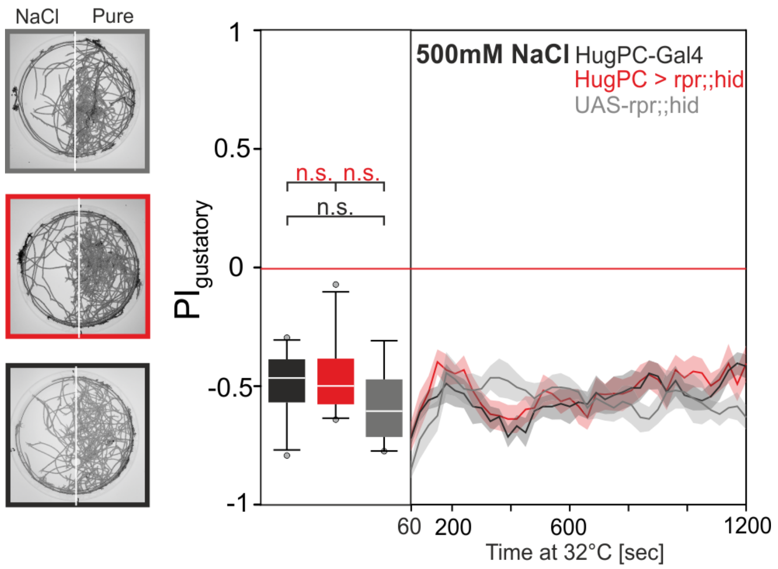

Supplementary Fig. 2: HuginPC are not necessary for salt avoidance

Two-Choice experiments on 500mM NaCl versus water substrate verify the lack of phenotype shown in Figure 5. Ablating huginPC neurons with UAS-rpr;;hid (n=11) led to no significant difference to the controls, HugPC-Gal4 (n=10, p= 0.944, Mann-Whitney-U-Rank-Sum-Test (MWU-Test)) and UAS-rpr;;hid (n=11, p=0.130, MWU-Test). Controls did not differ from each other (p=0.189, MWU-Test). Plates on the left show 5min time projections of the last 5 min of the 20 min long experiment. Significances are indicated as ***p<0.001, **p<0.01 and *p<0.05. Line plots showing the time course of the experiments are displayed as mean (line) ± SEM (transparent areas). Details of descriptive statistics and statistics against chance levels for experimental lines are shown in **Supplementary Table 7**.

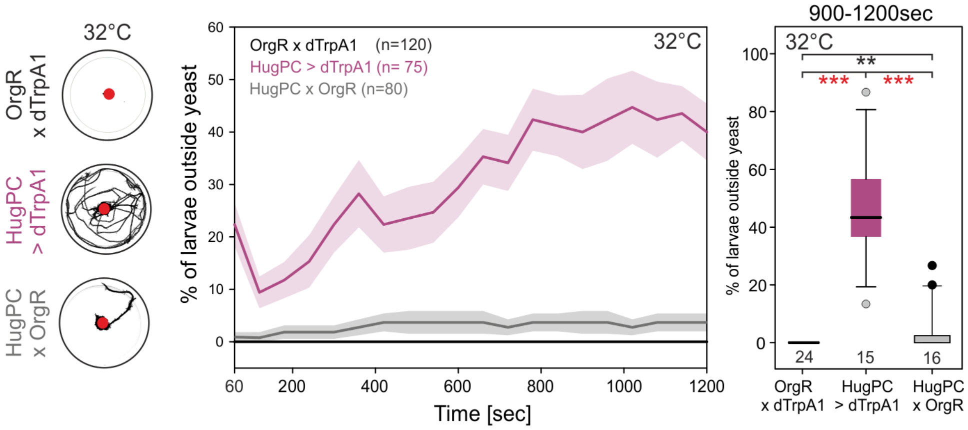

Supplementary Fig. 3: Activation of huginPC neurons causes wandering like phenotype

In the food intake assay larvae in which the huginPC neurons are activated (via UAS-dTrpA1 for 20 minutes) , leave a strongly attractive food source (yeast). Control larvae (OrgR x dTrpA1 and HugPC x OrgR) did not leave extensively the yeast (red spot in the middle of the petri dish) at any time point during the assay. The controls showed significantly less wandering behavior than HugPC>dTrpA1 larvae ($p<0.001$, Mann-Whitney-U-Rank-Sum-Test). Controls also differed from each other ($p=0.003$, MWU-Test). Numbers below boxplots represent number of petri dishes analyzed (5 larvae per dish). Significances are indicated as ***$p<0.001$, **$p<0.01$ and *$p<0.05$. Line plots showing the time course of the experiments are displayed as mean (line) ± SEM (transparent areas). Details of descriptive statistics are shown in **Supplementary Table 7**.

Supplementary Table 1: Descriptive statistics of Figure 1

Green p-values indicate significance
Red p-values indicate no significance

$p \leq 0.05$ = * / $p \leq 0.01$ = ** / $p \leq 0.001$ = ***

Statistics to Fig. 1c **Hugin activation 200mM Caffeine (box plots)**

Experimental cross	Hugin-Gal4	Hugin > dTrpA1	OrgR > dTrpA1
n (plates)	11	10	10
mean	-0.6629	-0.3315	-0.7115
median	-0.6455	-0.3333	-0.7121
Std.err.	0.0352	0.0306	0.0208
Std. dev.	0.1167	0.0968	0.0657
one-sample signed rank against 0		0.002	

Statistics to Fig. 1d **Hugin loss of function 200mM Caffeine (box plots)**

Experimental cross	UAS-rpr;;hid	Hugin > rpr;;hid	Hugin-Gal4	Hugin > shi^{TS}	UAS-shi^{TS}
n (plates)	14	10	10	10	10
mean	-0.7056	-0.2685	-0.6667	-0.3217	-0.7552
median	-0.7303	-0.3061	-0.6485	-0.3485	-0.7515
Std.err.	0.0299	0.0591	0.0346	0.0668	0.0161
Std. dev.	0.1118	0.1868	0.1147	0.2114	0.0508
one-sample signed rank against 0		0.006		0.002	

Supplementary Table 2: Descriptive statistics of Figure 2

Statistics to Fig. 2a **Hugin activation 2M NaCl (box plots)**

Experimental cross	Hugin-Gal4	Hugin > dTrpA1	OrgR > dTrpA1
n (plates)	10	11	10
mean	-0.8497	-0.1466	-0.7710
median	-0.8697	-0.1333	-0.8199
Std.err.	0.0295	0.0450	0.0462
Std. dev.	0.0933	0.1494	0.1461
one-sample signed rank against 0		0.019	

Statistics to Fig. 2b **Hugin ablation 2M NaCl (box plots)**

Experimental cross	Hugin-Gal4	Hugin > rpr;;hid	UAS-rpr;;hid
n (plates)	10	10	10
mean	-0.8497	-0.8361	-0.82
median	-0.8697	-0.8848	-0.8545
Std.err.	0.0295	0.0291	0.0213
Std. dev.	0.0933	0.0919	0.0674
one-sample signed rank against 0		0.002	

Statistics to Fig. 2c **Hugin activation 1M Fructose (box plots)**

Experimental cross	Hugin-Gal4	Hugin > dTrpA1	OrgR > dTrpA1
n (plates)	13	11	11
mean	0.7066	0.0533	0.693
median	0.7697	0.0600	0.693
Std.err.	0.0311	0.0222	0.002
Std. dev.	0.1120	0.0737	0.082
one-sample signed rank against 0		0.083	

Statistics to Fig. 2d **Hugin ablation 1M Fructose (box plots)**

Experimental cross	Hugin-Gal4	Hugin > rpr;;hid	UAS-rpr;;hid
n (plates)	10	12	10
mean	0.6810	0.6584	0.7764
median	0.7033	0.6273	0.8030
Std.err.	0.0455	0.0317	0.0251
Std. dev.	0.1439	0.1097	0.0793
one-sample signed rank against 0		<0.001	

Supplementary Table 3: Descriptive statistics of Figure 4

**Statistics to Fig. 4c**	**HugPC ablation 200mM Caffeine (box plots)**		
Experimental cross	HugPC-Gal4	HugPC > rpr;;hid	UAS-rpr;;hid
n (plates)	10	13	14
mean	-0.8345	-0.3981	-0.7056
median	-0.8367	-0.4788	-0.7303
Std.err.	0.026	0.0559	0.0299
Std. dev.	0.0837	0.2135	0.1118
one-sample signed rank against 0		0.004	

**Statistics to Fig. 4d**	**HugPC ablation 2M NaCl (box plots)**		
Experimental cross	HugPC-Gal4	HugPC > rpr;;hid	UAS-rpr;;hid
n (plates)	10	10	10
mean	-0.7509	-0.6376	-0.82
median	-0.7818	-0.6697	-0.8545
Std.err.	0.043	0.0420	0.0213
Std. dev.	0.1360	0.1379	0.0674
one-sample signed rank against 0		<0.001	

**Statistics to Fig. 4e**	**HugPC ablation 1M Fructose (box plots)**		
Experimental cross	HugPC-Gal4	HugPC > rpr;;hid	UAS-rpr;;hid
n (plates)	10	10	10
mean	0.6667	0.7194	0.7764
median	0.6515	0.6939	0.8030
Std.err.	0.0201	0.0402	0.0251
Std. dev.	0.0824	0.1271	0.0793
one-sample signed rank against 0		0.002	

Supplementary Table 4: Descriptive Statstics of **Figure 5**

Statistics to Fig. 5a **HugPC activation - food intake (box plots)**

Experimental cross	OrgR x dTrpA1	HugPC > dTrpA1	HugPC x OrgR
n (larvae)	120	75	80
mean fold change	2.3738	0.4515	2.4567
median fold change	2.3767	0.1966	2.6939
Std.err.	0.0532	0.0636	0.1511
Std. dev.	0.5823	0.5511	1.3511

Statistical tests described in the figure 5a were performed comparing the mean fold changes.

Statistics to Fig. 5b **HugPC activation – AN nerve recordings (box plots)**

Experimental cross	OrgR x dTrpA1	HugPC > dTrpA1	HugPC x OrgR
n larvae (n temperature steps)	9(32)	13(27)	10(26)
mean fold change/relative fold change	2.0490 / 0	1.4147 / -0.6343	2.1191 / 0.0701
median fold change/relative fold change	2.0109 / -0.0381	1.0123 / -1.0367	2.0378 / -0.0112
Std.err.	0.1688	0.2599	0.0701
Std. dev.	0.9550	1.4932	1.1807

Statistical tests were performed comparing the mean fold changes. Relative fold changes are displayed as boxplots in Fig. 5b!

Supplementary Table 5: Descriptive statistics of Figure 6

Statistics to Fig. 6c HugPC neurons - CaMPARI (box plots)

Taste substance	Water	20mM Caffeine	2M NaCl	1M Fructose	10% yeast	no UV light
n (larvae)	19	20	17	19	15	15
mean	0.7931	1.4093	0.5609	0.5419	0.4566	0.1847
median	0.7232	1.4816	0.5445	0.4944	0.4399	0.1870
Std.err.	0.0569	0.1373	0.0342	0.0387	0.0299	0.0122
Std. dev.	0.2481	0.6138	0.1366	0.1685	0.1305	0.0472
p-values		0.003	0.004	<0.001	<0.001	<0.001

Statistics to Fig. 6d HugPC neurons – Caffeine concentrations - CaMPARI

Taste substance	Water	10mM	20mM	50mM	100mM	200mM
n (larvae)	19	18	20	19	19	17
mean	0.7931	1.4236	1.4093	2.1257	2.4236	2.4904
Std.err.	0.0569	0.1310	0.1373	0.1891	0.0916	0.1651
p-values		<0.001	0.003	<0.001	<0.001	<0.001

Statistics to Fig. 6e HugPC - other bitter substances – CaMPARI (box plots)

Experimental cross	Water	10mM Quinine	10mM Denatonium
n (larvae)	19	15	15
mean	0.7931	1.3247	1.5921
median	0.7232	1.2527	1.4228
Std.err.	0.0569	0.1055	0.2030
Std. dev.	0.2481	0.4087	0.7863

Statistics to Fig. 6f HugPC ablation 10mM Denatonium (box plots)

Experimental cross	HugPC-Gal4	HugPC > rpr;;hid	UAS-rpr;;hid
n (plates)	10	10	12
mean	-0-6412	-0.1073	-0.4141
median	-0-5818	-0.0424	-0.3152
Std.err.	0-0570	0.0663	0.0557
Std. dev.	0-1802	0.2096	0.1931
one-sample signed rank against 0		0.160	

Supplementary Table 6: Descriptive statistics of Figure 7

Statistics to Fig. 7b GR66a activation – HugPC calcium imaging – peaks/min (bar plots)

Experimental cross	20°C control	30°C control	20°C experiment	30°C experiment
n (larvae)	13	13	14	14
mean	1.6035	1.8941	1.8893	5.0571
Std.err.	0.5730	0.7254	0.3553	0.3372

Supplementary Table 7: Descriptive statistics to Supplementary Figure 2 and 3

Statistics to Supplementary Fig. 2 HugPC ablation 500mM NaCl (box plots)

Experimental cross	HugPC-Gal4	HugPC > rpr;;hid	UAS-rpr;;hid
n (plates)	11	10	11
mean	-0.4937	-0.46	-0.5753
median	-0.4667	-0.5	-0.6061
Std.err.	0.0446	0.0526	0.0485
Std. dev.	0.1478	0.1665	0.1609
one-sample signed rank against 0		0.002	

Statistics to Supplementary Fig. 3 HugPC activation – % larvae leaving yeast (5 per plate) (box plots)

Experimental cross	OrgR x dTrpA1	HugPC > dTrpA1	HugPC x OrgR
n (plates)	15	24	16
mean	0	47.3333	3.8333
median	0	43.3333	-
Std.err.	0	5.1249	1.7816
Std. dev.	0	19.8486	7.9674
